# Nursing home finances associated with real estate investment trust and private equity investments

**DOI:** 10.1093/haschl/qxae037

**Published:** 2024-04-15

**Authors:** Dunc Williams, Rahul Fernandez, David Stevenson, Mark Unruh, Robert Tyler Braun

**Affiliations:** Department of Healthcare Leadership and Management, Medical University of South Carolina, Charleston, SC 29425-9620, United States; Center for Telehealth—Telehealth Center of Excellence, Medical University of South Carolina, Charleston, SC 29425-9620, United States; Population Health Sciences, Division of Health Policy and Economics, Cornell University, New York, NY 10065, United States; Department of Health Policy, Vanderbilt University Medical Center, Nashville, TN 37232, United States; The Geriatric Research, Education and Clinical Center (GRECC), Veterans Affairs Medical Center, Tennessee Valley Healthcare System, Nashville, TN 37212-2637, United States; Population Health Sciences, Division of Health Policy and Economics, Cornell University, New York, NY 10065, United States; Population Health Sciences, Division of Health Policy and Economics, Cornell University, New York, NY 10065, United States

**Keywords:** real estate investment trust (REIT), private equity, healthcare finance, nursing home

## Abstract

In 2021, real estate investment trusts (REITs) and private equity (PE) held investments in 1915 (16%) and 1569 (13%) US nursing homes (NHs), respectively. We created a database of REIT and PE investments in NHs, merged it with Medicare Cost Report data (2011–2019), and used a difference-in-differences approach within an event-study framework to compare NH spending and financial performance before and after REIT or PE investment to NHs that did not receive REIT or PE investment. REIT investments were associated with higher total wages (3%), total nursing wages (3%; both logged, per resident day [PRD]), and current ratio (81%). PE investments were associated with lower net patient service revenue (7%), total expenses (7%), and total wages (8%; all logged, PRD). The impact of REIT and PE investments in NHs may vary in different market conditions, as may occur in the current environment of low, falling NH profits, potentially higher minimum staffing requirements, and rising interest rates. Therefore, it is important for stakeholders to understand the impact of these large, growing investments on the financial performance of NHs.

## Introduction

Nursing home (NH) funding from institutional investors, such as real estate investment trusts (REITs), private equity (PE) funds, and for-profit entities (public and private) is significant and has grown in recent decades,^1-4^ yet much is unknown about how those investments impact NH financial performance. Nursing home financial performance is important to understand because of its potential association with quality of care, nurse staffing, organizational solvency, and access to NH care for the growing Medicare population.

Using a dataset identifying REIT and PE investments in NHs nationally,^2^ we investigated the financial performance of NHs with and without institutional investments. Specifically, we evaluate whether REIT and PE investments were associated with changes in profits (total and operating margins), wage costs (total and nursing), and liquidity (current ratio).

### REIT and PE investments

In the United States, REIT and PE investments have expanded significantly in recent years. These investments exist in many diverse retirement portfolios (eg, many mutual funds, index funds, and state pensions invest in REITs and PE).

REITs were created by Congress in 1960 to help individuals passively invest in real estate.^5^ The value of REITs in public markets grew from $1.5 billion in 1971 to $1.3 trillion in 2002, with 8% ($100 billion) in health care investments. Overall, REIT investments, including both public and private sectors, amounted to $4.5 trillion.^6^ For perspective, all public US companies were worth $41 trillion at the end of 2022.

Over the past 50 years, a series of US regulatory changes have increased opportunities for PE investments.^7^ Aiming for higher returns, PE firms often buy companies using borrowed money and ask investors to commit funds for 3 to 7 years.^8^ Private equity firms aim to sell investments within predetermined times, seeking high returns to reward investors for their assumed risk. Private equity market caps grew from $97 billion in 2000 to $1.1 trillion in 2021.^9^ Health care's share of PE investments has also risen, from less than 5% ($5 billion) in 2000 to over 14% ($100 billion) by 2018.^10^ Private buyers of NHs are estimated to have increased from 33% of all buyers in 2015 to 89% in 2021.^11^

Prior work suggests that, relative to not-for-profit owners, for-profit NH ownership is associated with lower quality post-acute and long-term care^12^; yet, modern evidence for PE and REIT investments is limited and has largely not been quantified. Research investigating the association of PE investments with NH quality and staffing has shown mixed results.^2-4,13-16^ An analysis of PE investments associated with NH profits for 2000–2007 found higher profits, revenue, and costs, but no change in payer mix.^17^ For REITs, evidence on nurse staffing is mixed.^1^

### Nursing home demand, financial performance, and institutional investor relationships

Nursing homes are crucial access points for aging Americans. Over half will eventually use an NH for either short-term post-acute or long-term care.^12,18^ As the US population ages, NH care demand is rising. From 2010 through 2020, the population aged 65+ years grew by 39%, 5 times faster than the overall population.^19^ By 2030, 1 in 5 Americans is projected to be age 65+ years.^20^ Nursing home spending is projected to grow from $166 billion in 2017 to $240 billion by 2025.^3^ This steady and growing demand might appeal to institutional investors.

Nursing home financial performance is complex. Between 2011 and 2019, reported profits were near zero and declined over time.^21^ Profits can vary for reasons like payer mix, with Medicare reimbursement for residents receiving post-acute care being more profitable than Medicaid reimbursements for those receiving long-term care.^22^ Nursing homes are asset-heavy organizations with much of their value attributed to real estate and facilities. In 2019, NH real estate was valued at $117 billion.^4^ In an environment of declining profits, many NH operators have sought opportunities to improve their cash position and profitability through relationships with outside investors (eg, REITs and PE). These investments take various forms, including property purchase leasebacks, management contracts, and acquisitions, and they often make complex financial relationships through related party transactions that are difficult to identify and evaluate.

Real estate investment trust and PE investors may target NHs because of the value of their real estate, predictable patient demand and volume, and consistent Medicare and Medicaid reimbursements.

Real estate investment trust and PE investments may improve financial performance at NHs by enhancing economic efficiencies (eg, leveraging group purchasing opportunities, scaling infrastructure resources like information technology, and reducing cash-holding requirements) and infusing capital for things like new technology, facility renovations, increasing staffing levels, or hiring more capable staff. Nursing home operators can benefit by selling physical assets to an REIT and then renting them back. This approach increases the operator's cash on-hand from the sale and transfers the liability of maintaining those assets.

Some institutional investments in NHs may be the result of financial distress. For example, an NH operator may sell to an REIT or PE firm to prevent closure. For investors, it may be an opportunity to invest at a discounted valuation, if they believe they can stabilize or improve operations.

Alternatively, REIT and PE investments may negatively impact NHs. To generate profits, 1 area to reduce costs may be staffing, which represents the highest proportion of expenses for the average NH.^21^ Reduced NH staffing has been associated with poorer quality performance measures.^1,14,23-25^ Further, increasing costs of capital in the current environment of rising interest rates may negatively impact the benefits of these relationships for both NHs and investors over time. For NHs receiving REIT investments, the benefit may diminish over time if lease rates rise while NH profits remain stagnant. For NHs receiving PE investments, there may be various impacts on long-term performance, particularly if the proportion of debt increased significantly during the PE investment.

In this study we evaluate financial measures associated with costs, quality (which is impacted by wage expenses),^1,14,23-25^ and access (eg, financially fragile facilities may provide less care, or ultimately close).

## Data and methods

### Data

Using multiple data sources, we created a panel dataset of NH characteristics, including REIT and PE investments, for the period 2011–2019. We used previously developed approaches to identify PE^2,12^ and REIT^1^ investments in NHs using data from the Centers for Medicare and Medicaid Services (CMS) Provider Enrollment, Chain, and Ownership System (PECOS), S&P Capital IQ, and Irving Levin (IL) Associates Health Care M&A databases, supplemented by web-based searches. Private equity firms acquire nursing homes and REITs conduct sale leasebacks; both transactions are collectively termed “investments.” Detailed information on the methods used to identify REIT and PE investments can be found in prior studies.^1,2,12^

We combined NH characteristics from LTCFocus and several CMS databases: PECOS, the NH Compare (NHC) Ownership File, and the CMS Hospital Cost Report Information System (HCRIS) “Cost Reports” (Appendix 1).

### Study sample

Our study sample consisted of all for-profit NHs that met our inclusion criteria for 2011–2019 (Appendix 2). We began with NHs reporting Cost Reports between 2011 and 2019. We excluded NHs that reported information for less than 360 days and more than 370 days in a period, were in US territories, and were not-for-profit. We created 2 treatment groups for analyses. The first group included NHs that received REIT investments over the period 2012–2018. The second group included facilities that received PE investments over the same period. The NHs in our treatment groups were limited to those that received REIT or PE investments between 2012 and 2018 (referred to as “REIT NHs” and “PE NHs”) to allow comparisons of outcomes to NHs in the comparison groups (“non-REIT” or “non-PE” NHs) for at least 1 year before and at least 1 year after REIT or PE investment. The non-REIT and non-PE NHs in our comparison groups consisted of for-profit NHs that never received REIT or PE investments, respectively, during the sample period. If an NH received investment from more than 1 REIT or PE firm during our study period, we only considered the first investment.

### Study design

In separate analyses, we used a difference-in-differences (DID) approach within an event-study framework to compare changes in financial measures after REIT and PE investments to concurrent changes in NHs without REIT or PE investment. Recent econometric research has shown that DID estimates can be biased if treatment effects vary over time or if multiple treatment periods and groups are defined by when the treatment took place.^26^ Due to these concerns, we used a DID estimator within an event-study framework developed to address these issues (eg, Callaway and Sant'Anna^27^). The control group included never-treated observations. We used inverse probability of treatment weights because we theorize that the treatment model is correctly specified. An assumption required for the DID approach is that preacquisition differences between the PE/REIT and control NHs would remain constant in the absence of a PE acquisition. To test this assumption and to show changes over time, we conducted an event-study analysis.

Standard errors were adjusted for clustering at the NH level. The unit of analysis was the NH-year. We also conducted a descriptive portion of the study to examine the means and proportions of NH facility characteristics and outcomes.

In sensitivity analyses, we accounted for unobserved correlation of year-specific effects between model variables and REIT or PE investments (eg, implementation of various CMS or state-level policies) with year and region fixed effects in our DID models.

### Study variables

#### Outcome measures

Outcome measures used to assess NH financial performance included profits (total and operating margins), net patient service revenue (NPSR) per resident day (PRD), total expenses PRD, wage costs (total and total nursing) PRD, and liquidity (current ratio). All dollar-value measures (NPSR, expenses, and wage costs) were logged to account for skewed distributions.

##### Profitability

Profitability was measured with total margin and operating margin. Total margin is important because it measures the overall profitability of an organization. Operating margin is important because it measures the profitability of an organization's core services. Higher profits reflect better financial performance. Profits are critical to organizational long-term sustainability. Revenue was measured with NPSR PRD; expenses were measured by total expenses PRD.

##### Wage costs

Wage costs were measured with total nursing and total wages, both PRD. Clinical nursing variables measure NH investment in direct patient care staffing,^28^ an area receiving much attention from CMS, including a recently proposed rule to increase national minimum staffing requirements.^29^ Total wages, comprising the largest of NH expenses (>40% in 2019),^21^ measures the proportion of spending on wages. Both measures were for employed, non-contract labor.

##### Liquidity

Liquidity was measured with the current ratio. Current ratio was calculated as current assets divided by current liabilities. A current ratio above 1.0 suggests an organization has enough current assets to meet upcoming debt obligations. While profitability measures are paramount to understand organizational long-term sustainability, liquidity measures are critical to assess organizational capacity to meet near-term obligations. At times, organizations will display liquidity concerns before filing for bankruptcy (eg, an inability to clear employee paychecks due to insufficient cash^30^ may occur before a bankruptcy filing).

#### Key explanatory variables

For the REIT analysis, the key explanatory variable was a binary measure for whether an NH received REIT investment. Separately in the PE analysis, the key explanatory variable was a binary measure for whether an NH received PE investment.

#### Other explanatory variables

Other explanatory variables included NH and aggregated resident characteristics. Nursing home characteristics consisted of occupancy rate, total beds, and percentages of Medicare and Medicaid residents. Resident characteristics included age, case-mix index, percentage non-White (due to a large number of redacted observations in LTCFocus), and percentage female. Case mix was measured using the average resident acuity index derived from Minimum Data Set assessments, which reflects the level of care needed by residents. A higher index value indicates greater needs (scores range from 0 to 24).

### Limitations

Our study has limitations. First, REIT and PE investments in NHs are complex, multilayered, and not easy to determine. We identified REIT and PE NH investments using S&P Capital IQ and IL, which rely on public announcements and may not include smaller investments or investments that have nondisclosure agreements. This may bias our estimates toward no effect. However, for PE investments, we found a similar number of NH acquisitions as other studies.^2^ Second, we mainly identified publicly listed REITs; we likely did not capture all privately listed REITs. Third, the PECOS file may not always identify the REIT as an owner. In this case, REIT NHs may appear to be without REIT investment. Fourth, the approach we used to identify operators likely resulted in an underestimate of affiliates due to inaccurate and missing observations that occur at random in the cost report data.^32,33^ Fifth, while REITs typically make long-term investments, we did not investigate divestitures and our sample was limited to the first investment during the study period. Sixth, LTCFocus measures are based on the calendar year, and may not contemporaneously reflect Cost Report outcomes, which are based on the fiscal year. Seventh, Cost Reports have been shown to report certain data inconsistently.^31^ However, they have been widely used for research and are the most comprehensive source of information on financial performance.^32^

## Results

### REIT and PE investment activity

In 2021, 1915 (16%) NHs had REIT investment and 1569 (13%) had PE investment (Appendix 3). In 2021, 77% of our NH sample were for-profits; 23% were nongovernmental not-for-profits.

### Nursing home characteristics

In unadjusted comparisons presented in Table 1, REIT NHs had the highest percentage of Medicare residents, total margin, operating margin, NPSR, expenses, and total wages followed by PE, then other for-profit NHs. The PE NHs had the highest total nursing wages PRD, followed by REIT, then other for-profit NHs.

**Table 1.** Characteristics of nursing homes by for-profit (control), REIT, or PE association in the pooled sample (2011–2019).

	2011–2019 Pooled sample		
Characteristics	For-profit (*n* = 5138)	REIT (*n* = 301)	PE (*n* = 124)
Nursing home characteristics			
Medicare residents, %	14.05	18.93	16.94
Medicaid residents, %	62.93	57.45	64.14
Occupancy rate, %	79.83	77.96	83.63
Chain facility, %	54.65	82.97	87.11
Total beds, mean	108.62	101.72	107.99
Resident characteristics			
Age, mean, y	78.61	78.79	78.39
Non-White, %	21.35	21.78	22.23
Female, %	65.84	64.45	65.80
Acuity index, mean	12.08	12.11	12.38
Outcomes			
Total margin, %	0.49	2.02	(0.09)
Operating margin, %	(0.52)	0.82	(0.72)
Net patient service revenue per resident day, mean	$276.18	$341.90	$325.34
Total expenses per resident day, mean	$286.16	$347.54	$339.13
Total wages per resident day, mean	$121.52	$143.88	$134.15
Total nurse wage costs per resident day, mean	$68.85	$75.98	$ 82.33
Current ratio, mean	2.02	1.96	1.85

Abbreviations: PE, private equity; REIT, real estate investment trust.

Source: Authors’ analysis of Cost Report and LTCFocus data from 2011–2019. For-profit includes nursing homes that never received REIT or PE investment during the sample period.

We excluded homes where we determined REITs or PE divested before 2018, invested before 2012, or invested after 2018.

### Changes in outcomes after REIT investment

The DID results for REIT and PE investments are shown in Table 2. REIT investment was associated with higher total wages (3%), total nursing wages (3%) (both logged, PRD), and current ratio (81%). Profit, revenue, and total expense estimates were not statistically significant, although there were some yearly differences in outcomes associated with REIT investment in the event-study estimates.

**Table 2.** Changes in financial outcomes after REIT or PE investment compared with for-profit nursing homes without PE or REIT ownership.

	REIT (*n* = 2507)		PE (*n* = 977)	
Outcome	REIT pooled sample, 2011–2019	ATT	PE pooled sample, 2011–2019	ATT
Total margin (%)	0.59	−0.35	0.54	0.78
Operating margin **(%)**	−0.44	−0.54	−0.68	0.71
log Net patient service revenue ($) per resident day	280.42	0.01	298.31	−0.07***
log Total expenses ($) per resident day	290.10	0.01*	310.05	−0.07***
log Total wages ($) per resident day	122.96	0.03***	129.65	−0.08***
log Total nurse wage costs ($) per resident day	69.31	0.03***	73.61	0.01
Current ratio	2.01	0.81***	1.97	0.10

Abbreviations: ATT, average treatment effect on treated; PE, private equity; REIT, real estate investment trust.

Source: Authors’ analysis of Cost Report and LTCFocus data from 2011–2019. Estimates generated from Callaway and Sant'Anna^27^ difference-in-differences estimator. Implements a difference-in-difference with multiple periods estimator to decompose a 2-way fixed-effects (TWFE) model with staggered treatment to individual 2 × 2 difference-in-differences estimations. Per resident day (PRD) was calculated by dividing the outcome dollars by 365 days.

***P < .01, *P < .1.

**Figure 1.**
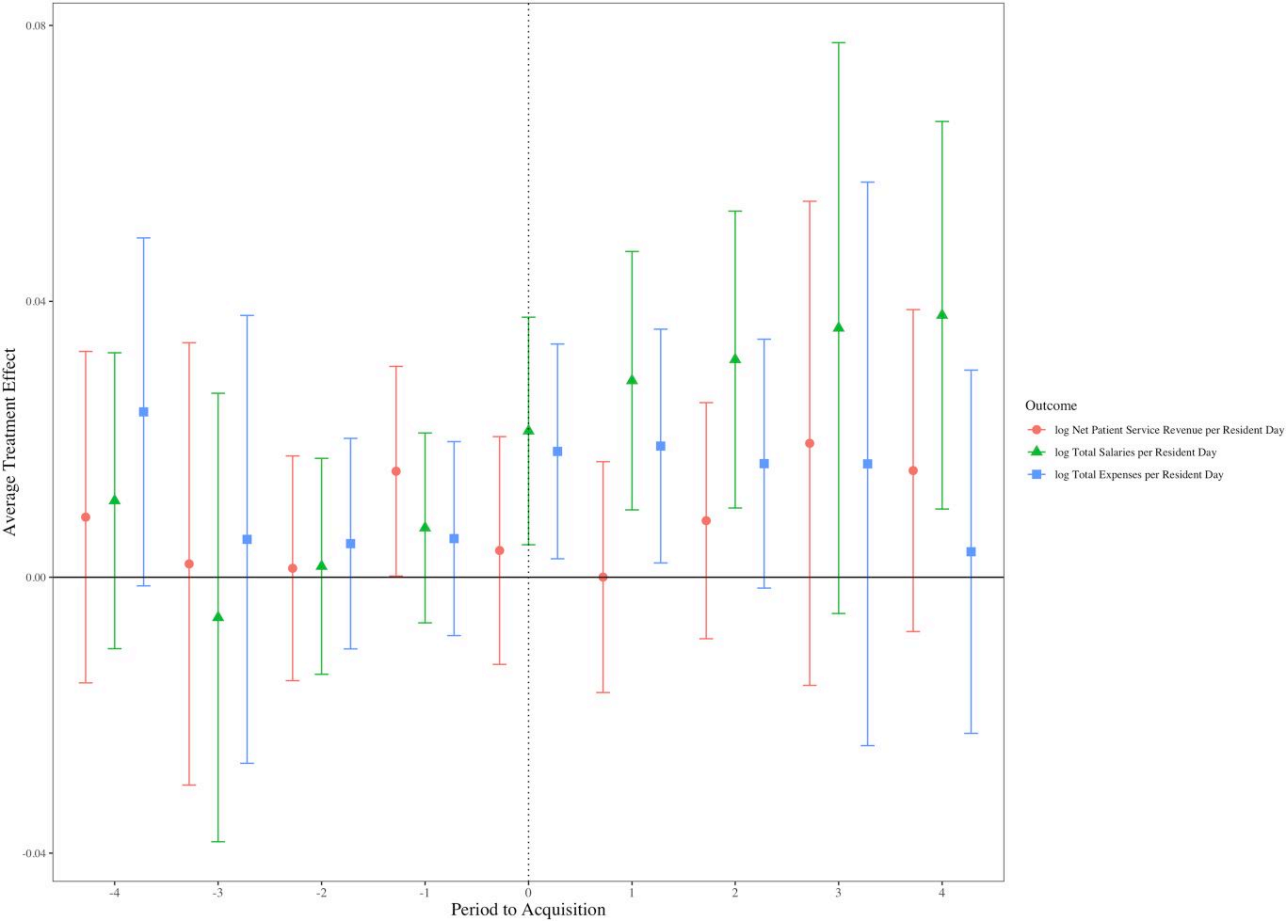
Association of REIT investment on nursing home finances by year, 2011–2019. Source: Authors’ analysis of Cost Report and LTCFocus data from 2011–2019. REITs are for-profit public or private corporations that invest in income-producing properties. The figure illustrates the difference in net patient service revenue, total expenses, and total wages (all per resident day) in REIT vs non-REIT nursing homes (sample sizes are shown in Table 1). Non-REIT nursing homes are for-profit homes that never received REIT investment. Estimates were generated from a Callaway and Sant'Anna^27^ difference-in-differences estimator. Abbreviation: REIT, real estate investment trust.

As presented in Figure 1, key REIT NH event-study plots show an increase in total expenses in year 1 and increases in total wages (years 1,2,4) (logged, PRD). In other REIT NH event-study plots, presented in Appendix 4, operating margin was lower (year 1), expenses were higher (year 1), and wages increased in some years for total wages (years 2–4) and total nursing wages (years 2–4). The current ratio for REIT NHs increased post-investment in years 2–4. The event-study estimates show changes in the outcome variables for NHs by year. All estimated coefficients from the event-study models in the period before acquisition did not differ significantly between the treatment and control NHs, demonstrating that the parallel trends assumption fundamental to DID analyses was not violated.

### Changes in outcomes after PE investment

**Figure 2.**
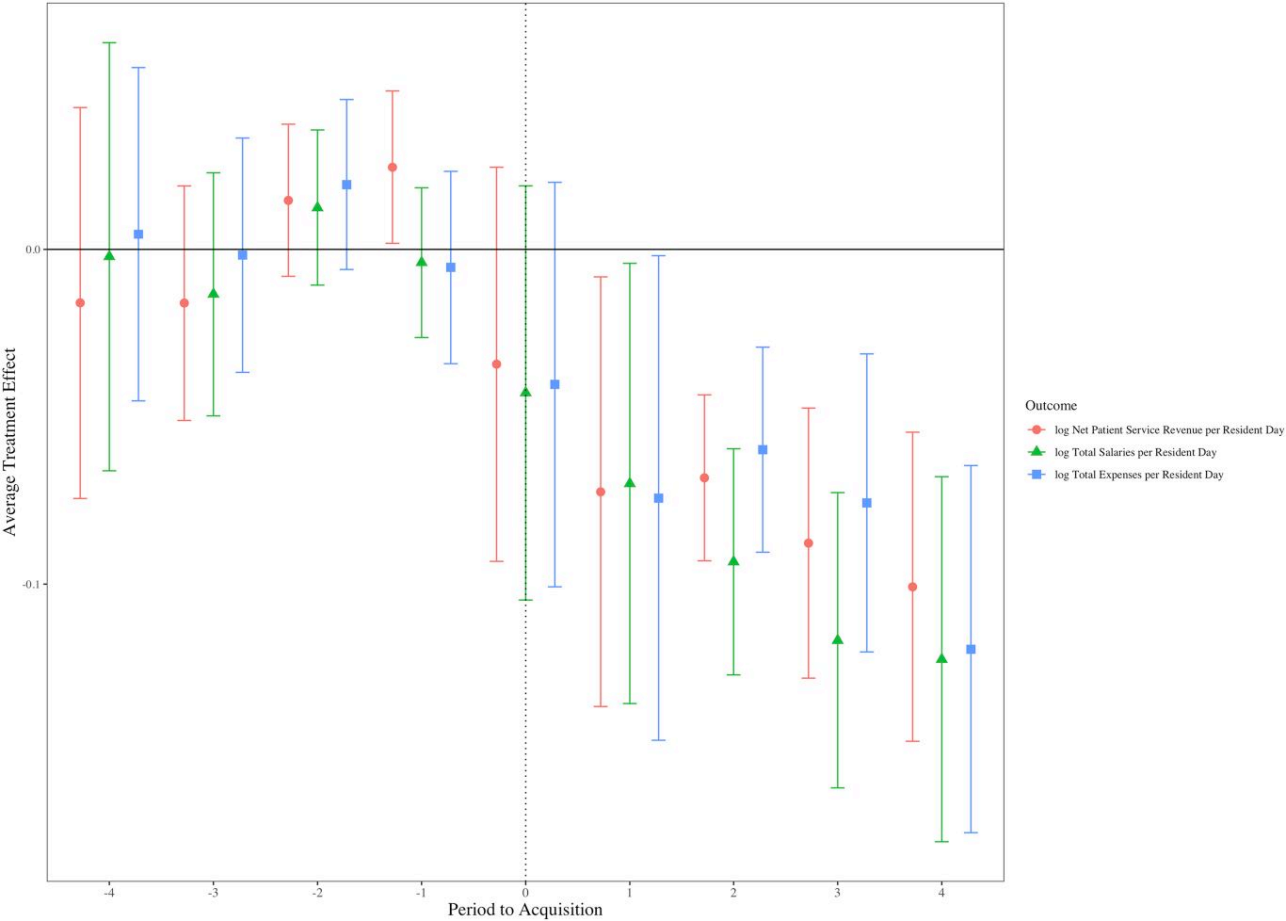
Association of PE investment on nursing home finances by year, 2011–2019. Source: Authors’ analysis of Cost Report and LTCFocus data from 2011–2019. The figure illustrates the difference in net patient service revenue, total expenses, and total wages (all per resident day) in PE vs non-PE nursing homes (sample sizes are shown in Table 1). Non-PE nursing homes are for-profit homes that never received PE investment. Estimates were generated from a Callaway and Sant'Anna^27^ difference-in-differences estimator. Abbreviation: PE, private equity.

For the study sample and period, both DID estimates (Table 2) and event-study plots (Figure 2) show that PE investment was associated with lower NPSR (7%), total expenses (7%), and total wages (8%) (all logged, PRD). In the DID analyses, profits, total nursing wages, and current ratio were not statistically significant.

### Sensitivity analyses

Appendix 5 includes discussion of additional outcomes related to nursing wage types (eg, registered nurses [RNs], licensed practical nurses [LPNs], and certified nursing assistants [CNAs]), total contract nurse wages, an additional measure of liquidity, days of cash on hand, and a measure of debt structure, long-term debt to capital. Of those, only REIT investment was associated with any statistically significant differences, specifically higher RN wages (5%) and CNA wages (4%).

Appendix 6 shows results for selection based on resident and facility characteristics. For REITs, only the estimate associated with the percentage of residents covered by Medicaid was statistically significant (1.61%); for PE, estimates associated with the percentage of Medicaid-covered residents (2.78%), percentage female (−1.02%), and percentage of White residents (1.46%) were statistically significant; however, the effects were relatively small.

Appendix 7 displays results accounting for unobserved correlation of year-specific effects between model variables and REIT or PE investments with year and region fixed effects; results were consistent with those from our primary analysis.

## Discussion

In this national study, REIT investment in NHs was associated with increases in total wages (3%), nursing wages (3%), and current ratio (81%). The PE investments in NHs were associated with a 7% decrease in both NPSR and total expenses, and an 8% decrease in total wages. Neither REIT nor PE investments were associated with changes in profits.

Real estate investment trust and PE investments impact overall financial performance of NHs. However, these investments did not appear to influence NH profits. For NHs receiving REIT investments, there was a notable rise in various nursing wages, hinting that these NH operators reinvested funds into labor, their biggest expense. This is consistent with an earlier study that found that more hours per resident day were allocated for LPN and CNA nurse staffing associated with the largest REIT deals.^1^ Taken together, NHs appear to have redeployed some returns from REIT investments into staff and wage increases. Conversely, PE-invested NHs experienced decreases in revenue, expenses, and total wages, but without any significant impact on profits. While PE appeared to target cost cuts, both overall and labor-specific, they experienced a dip in revenue, resulting in a net zero effect on profits.

For REIT-invested NHs, the current ratio significantly increased. This may occur for 2 reasons: (1) the REIT investment increased cash reserves, hence raising current assets, and (2) NH operators used cash to reduce debts, improving liquidity. Supporting this, our event-study (Appendix 4, Figure S4) revealed that the current ratio climbed in the following 2–4 years. Such a cash infusion may result in different actions by for-profit and not-for-profit NHs, as for-profits may be more likely to use cash to pay down debt or pay out dividends. Further, NH system affiliations may impact accounting practices and cash reserves held within specific subsidiaries.

### Implications

Our novel findings advance the literature on the impact of REIT and PE investments in NHs by examining financial performance and may benefit policymakers, NH residents, caretakers, NH operators, board members, providers, and investors.

Our wage findings may inform discussions about Medicaid payment allocations to wages and minimum NH staffing requirements. For example, CMS has proposed minimum staffing requirements,^29^ and MedPAC (Medicare Payment Advisory Commission) recently recommended increasing required staffing levels.^33^ Importantly, previous research has associated staffing levels with NH quality.^2,28,34-38^ The REIT NHs had higher wages, which may result from improved financial position associated with REIT investment. The PE-invested NHs had lower wages, which may result from efforts to reduce expenses and potentially improve efficiencies. Findings indicating that neither REIT nor PE investments impacted NH profits may suggest any managerial actions aimed at wages, revenue, or expenses resulting from these investments did not ultimately affect profits.

For an industry reporting declining, near-zero profits over the past decade, and generally relies on fixed reimbursements, regulators may be interested in monitoring financial impacts of REIT and PE investments on NHs. Rising inflation may result in higher lease expenses for REIT NHs, thus increasing expenses. Increasing interest rates may impact PE NHs, as the cost of debt rises and PE investments are often leveraged with debt.

If expenses rise at NHs, policymakers should be particularly interested because NHs are largely reliant on government reimbursements from Medicare and Medicaid. Table 1 shows that REIT NHs had the highest Medicare payer mix, followed by PE NHs, then other for-profits. While the hospital industry benefits from higher-than-cost commercial reimbursement cross-subsidization, the NH industry has much less commercial reimbursement^21^ and is largely cross-subsidized by Medicare reimbursement.^22^ Therefore, if inflationary factors notably increase costs at REIT NHs (through rent expenses) or PE NHs (through higher debt rates), government reimbursement at NHs may be particularly sensitive.

Antitrust regulators considering merger and investment oversight^39^ may be interested in our findings related to roll-up mergers involving the acquisition of several smaller facilities in a sector that may not, separately, trigger oversight.^40^

Our findings are relevant to CMS's recent final rule on NH ownership disclosures.^41^ The 1933 Securities Act, and several provisions since then, enhanced for-profit ownership transparency. Reporting requirements in CMS's recent final rule should not be overly burdensome and define criteria for types of investments (eg, ownership stake [and amount], affiliation type, etc). We evaluated Cost Report related-party transactions (unreported) and found no statistically significant associations. Future work on related-party transactions may improve our understanding of the financial condition of the NH industry. Opportunities to improve related-party transaction evaluations may exist through auditing NH financial data reported on Cost Reports, particularly worksheets A-8-1 and the G-series, or standardizing state-level Medicaid Cost Reports. Practical opportunities to annually file more transparent ownership/investment relationships, and dollar amounts, on existing required filings are the CMS-855A^41^ and Cost Reports.

For NH operators, the increase in cash at REIT NHs should be a benefit, at least in the near term. Contrarily, selling the NHs’ largest assets (eg, land, buildings) to increase cash position—and the subsequent need to lease those assets back long term—should be weighed. Longer-term, how cash is redeployed can affect organizational performance. For example, cash may be invested in other return-generating investments, used to pay down debt, or dispersed through various decisions, like increasing wages. Real estate investment trust NH operators should also consider impacts of REIT investment on organizational creditor ratings. Private equity investments in NHs, which our estimates indicate were not associated with a change in cash, may increase access to capital for the NH. Unlike REIT NHs, where REITs do not directly own the NH operator, PEs may acquire both the facility and the operator. Private equity ownership should be a stronger signal of capital access than cash held on hand at the NH because the PE parent organization holds cash it can use for the individual NH.

Cash management will be particularly important following the COVID-19 pandemic. We expect a long-term period of higher proportions of cash reserves held in response to organizational leaders recalling a pandemic-era need for cash. Such an occurrence may suppress industry growth for 2 reasons. First, cash generally yields lower returns than longer-term investments. Second, cash held requires financing (debt or equity)—and it is an asset that is not being used to grow business (eg, through expansions or acquisitions). While cash increased for REIT NHs, profits did not change.

For REIT NHs, the increase in wages suggests that operators used some proceeds from REIT investments to increase staff wages, which may be a goal for NH operators. No changes in total expenses may suggest that operators scaled non-wage efficiencies (eg, group supply purchasing or Internet technology [IT] services) through REIT affiliation in ways that reduced bottom-line expenses.

For PE NHs, we found lower total and wage expenses, similar to the magnitude of revenue reduction. This may indicate that PEs can enhance efficiencies but do so in ways that proportionally reduce revenue.

While REIT and PE investments in NHs are large and growing, the favorability of those investments may change in different market conditions, as may occur with low, falling NH profits, potentially higher minimum staffing requirements, and rising debt costs. Such a change may have unknown implications for the financing and financial performance of NHs.

## Conclusion

This study presents the first empirical evidence of REIT and PE investments on NH financial performance. Real estate investment trust NH investments may improve financial performance through higher total wages, nursing wages, and current ratio. Private equity NH investments may lead to lower revenue and total wages, but also lead to reduced expenses.

## Supplementary Material

qxae037_Supplementary_Data
