# Supplementary material for: Nursing home finances associated with real estate investment trust and private equity investments
Source: Health Aff Sch. 2024 Apr 15;2(4):qxae037. doi: 10.1093/haschl/qxae037 (PMC11034530; doi:10.1093/haschl/qxae037)
Supplement: qxae037_Supplementary_Data [file qxae037_supplementary_data.zip › Appendix Material 2_29_24 Clean Version.docx]

Table of Contents

[Appendix 1 – Cost Report Cleaning Process 2](#_Toc153289610)

[Appendix 2 – Study Sample Inclusion Criteria 2](#_Toc153289611)

[Appendix 2 Figure 1 – Inclusion Criteria for the Dataset 3](#_Toc153289612)

[Appendix 2 Figure 2 – Inclusion Criteria by REIT / PE Sample 4](#_Toc153289613)

[Appendix 3 – Mapping 2021 REIT and PE Investments by Hospital Referral Region (HRR) 5](#_Toc153289614)

[Appendix 3 Figure 1 – Map of 2021 REIT and PE Investments by HRR 6](#_Toc153289615)

[Appendix 4 – REIT Event Study Plots 7](#_Toc153289616)

[Appendix 4 Figure 1 – Association of REIT Investment on Nursing Home Profit Margins by Year, 2011-2019 8](#_Toc153289617)

[Appendix 4 Figure 2 – Association of REIT Investment on Nursing Home Revenue and Expenses by Year, 2011-2019 9](#_Toc153289618)

[Appendix 4 Figure 3 – Association of REIT Investment on Nursing Home Total Wages and Total Nursing Wages by Year, 2011-2019 10](#_Toc153289619)

[Appendix 4 Figure 4 – Association of REIT Investment on Nursing Home Current Ratio by Year, 2011-2019 11](#_Toc153289620)

[Appendix 5 – Sensitivity Analyses for Additional Outcomes 12](#_Toc153289621)

[Appendix 5 Table 1 – Additional Characteristics of Nursing Homes by For-Profit (Control), REIT, or PE Association in the Pooled Sample, 2011-2019 13](#_Toc153289622)

[Appendix 5 Table 2 – Sensitivity Analyses for Difference-in-Differences in Additional Outcomes After REIT or PE Investment Compared to For-Profit Nursing Homes without PE or REIT Ownership, 2011-2019 14](#_Toc153289623)

[Appendix 5 Figure 1 – Association of REIT Investment on Nursing Home RN, LPN, and CNA Wages by Year, 2011-2019 15](#_Toc153289624)

[Appendix 6 – Sensitivity Analyses for Selection Based on Resident and Facility 16](#_Toc153289625)

[Appendix 7 – Sensitivity Analyses Accounting for Year and State Fixed Effects 17](#_Toc153289626)

[Appendix References 19](#_Toc153289627)

# Appendix 1 – Cost Report Cleaning Process

Because NHs report to CMS based on organizational fiscal year (FY) instead of calendar year (CY), we associated cost reports to calendar years with the following attribution method. Those reporting a fiscal year end of January 1 – June 30 were attributed to the calendar year; those reporting a fiscal year end of July 1 - December 31 were attributed to the following year. For example, those reporting a fiscal year end of July 1, 2019 through June 30, 2020 were recognized as calendar year 2019 cost reports. This associated NH cost reports with the calendar year in which there were the most calendar days.

To account for outliers in cost report data, we Winsorized extreme values at the one percent tails of each variable’s distribution.(1, 2) Then, we tested and excluded values for each variable or ratio we considered impossible (e.g., greater than 100% Medicare payer mix) or improbable (e.g., an absolute value of total margin greater than 50%).

# Appendix 2 – Study Sample Inclusion Criteria

Appendix 2 Figure 1 shows the inclusion/exclusion criteria for the dataset. Appendix 2 Figure 2 shows the final exclusion criteria by REIT or PE investment.

## Appendix 2 Figure 1 – Inclusion Criteria for the Dataset


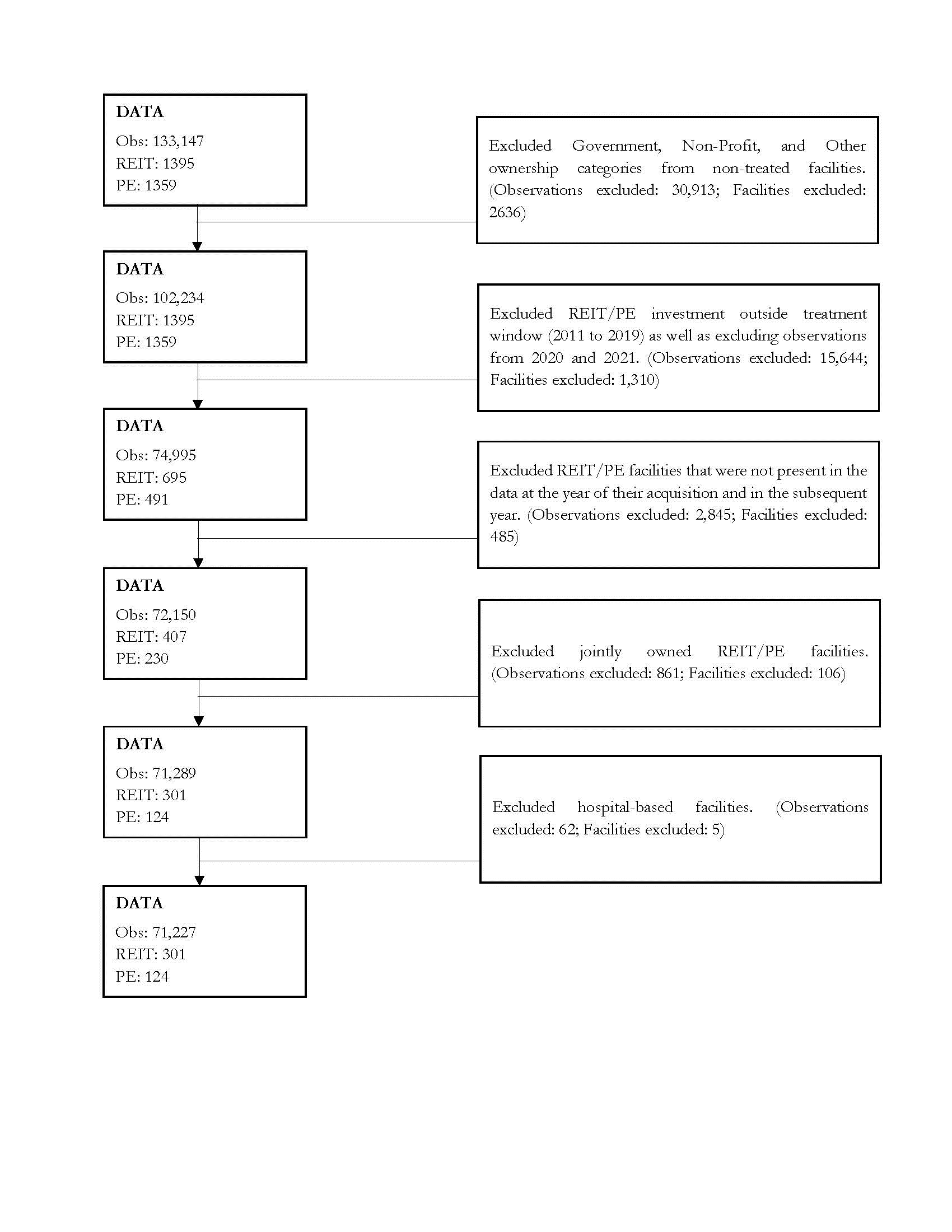


## Appendix 2 Figure 2 – Inclusion Criteria by REIT / PE Sample


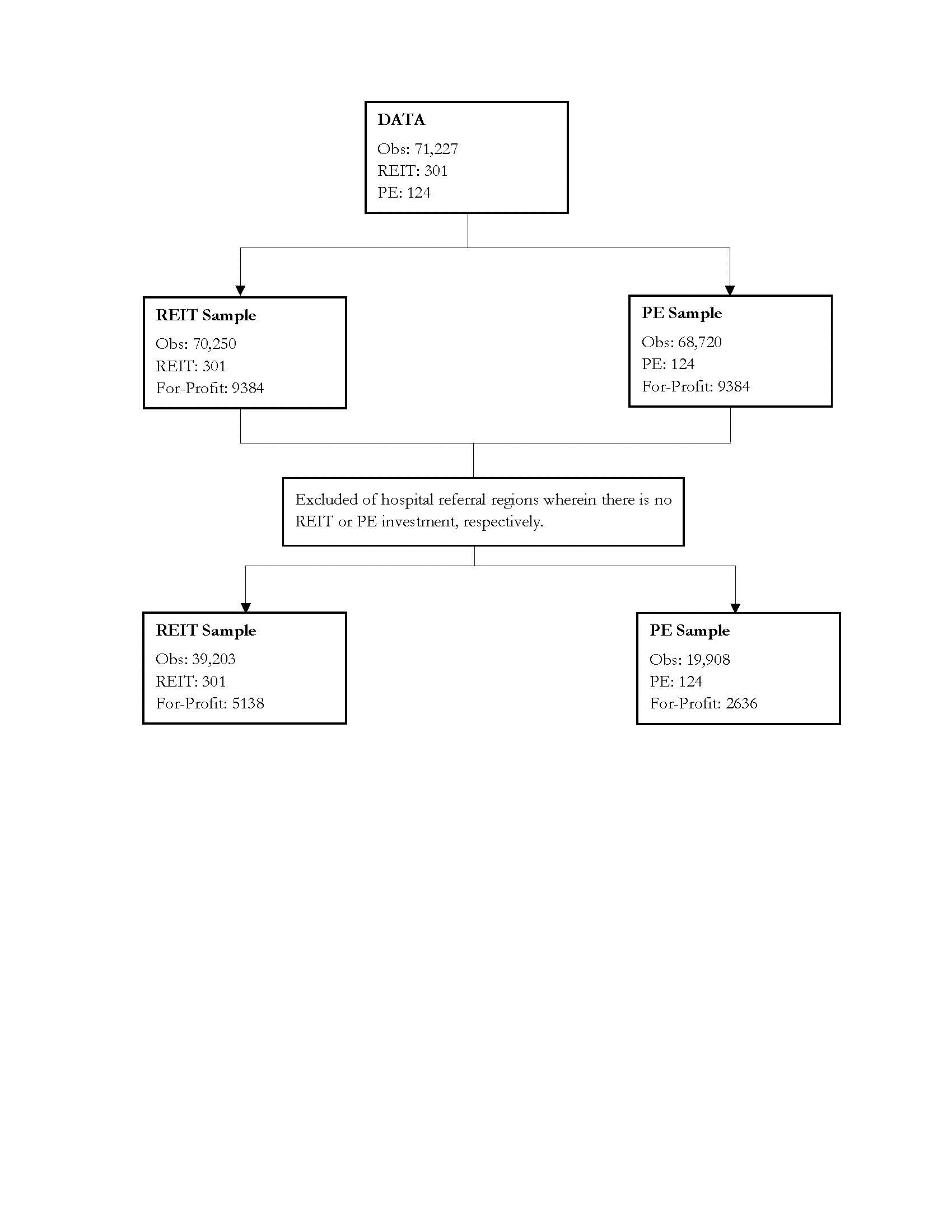


# Appendix 3 – Mapping 2021 REIT and PE Investments by Hospital Referral Region (HRR)

This map shows the geographical distribution and density of 2021 REIT and PE NHs by Hospital Referral Region (HRR). The mapped sample is based on our analytical sample, described in the main manuscript, which is smaller than the total number of REIT and PE NHs we identified and stated in the main manuscript.

# Appendix 3 Figure 1 – Map of 2021 REIT and PE Investments by HRR


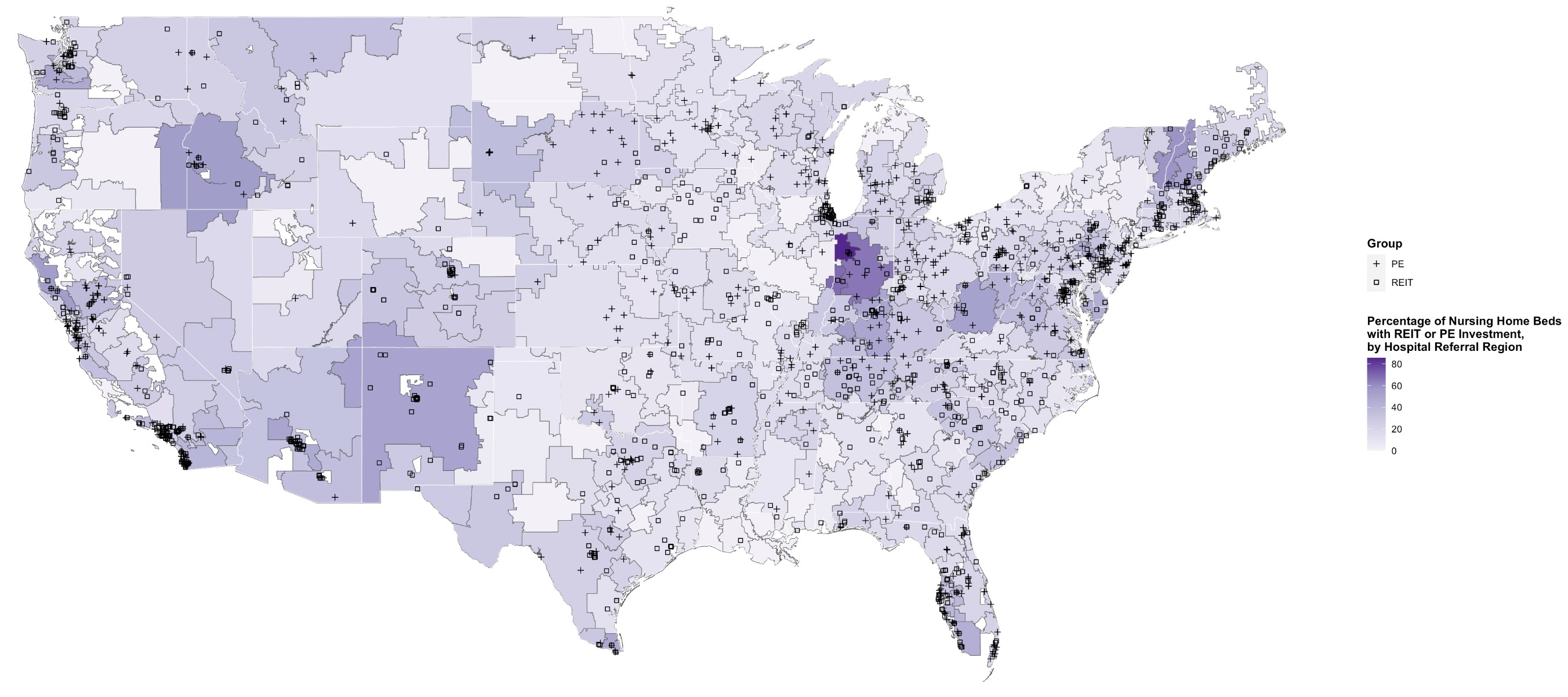


# Appendix 4 – REIT Event Study Plots

In Appendix 4 Figures 1-5, event-study approaches over time for REIT NHs show statistically significant differences across some years. Event-study plot estimates are displayed for a 4-year time frame both prior to and after the acquisition. The aim of this approach was to mitigate the influence of potential outliers, particularly in estimates that are distant from the period of investment.

Operating margin was lower (year 1, Appendix 4 Figure 1), expenses were higher (year 1, Appendix 4 Figure 2), wages increased in some years for total wages (years 2 – 4, Appendix 4 Figure 3), and total nursing (years 2 – 4, Appendix 4 Figure 4) wages. Appendix 4 Figure 4 shows current ratio decreased for REIT NHs in the year prior to investment, then increased post-investment in years 2-4.

## Appendix 4 Figure 1 – Association of REIT Investment on Nursing Home Profit Margins by Year, 2011-2019


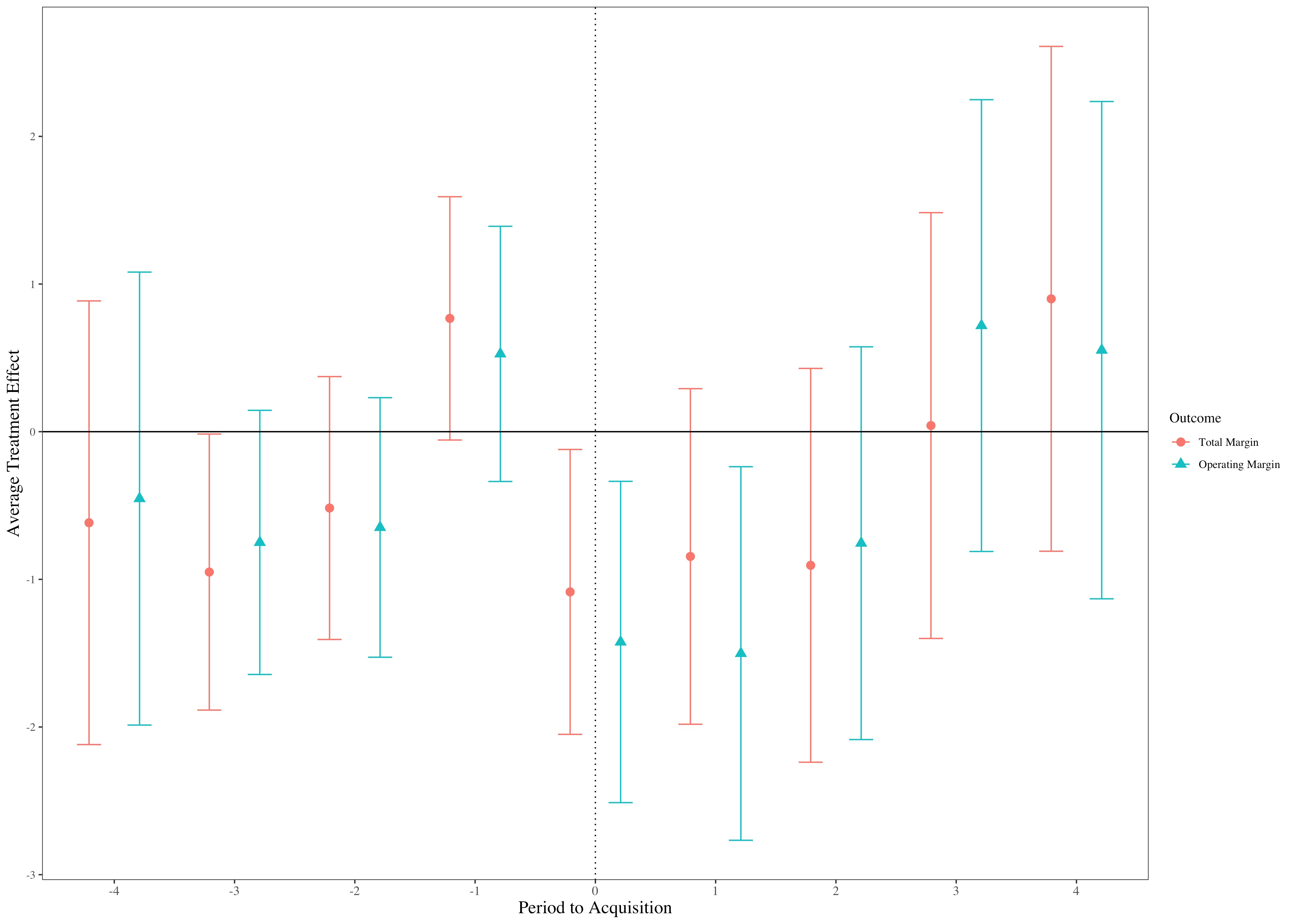


## Appendix 4 Figure 2 – Association of REIT Investment on Nursing Home Revenue and Expenses by Year, 2011-2019


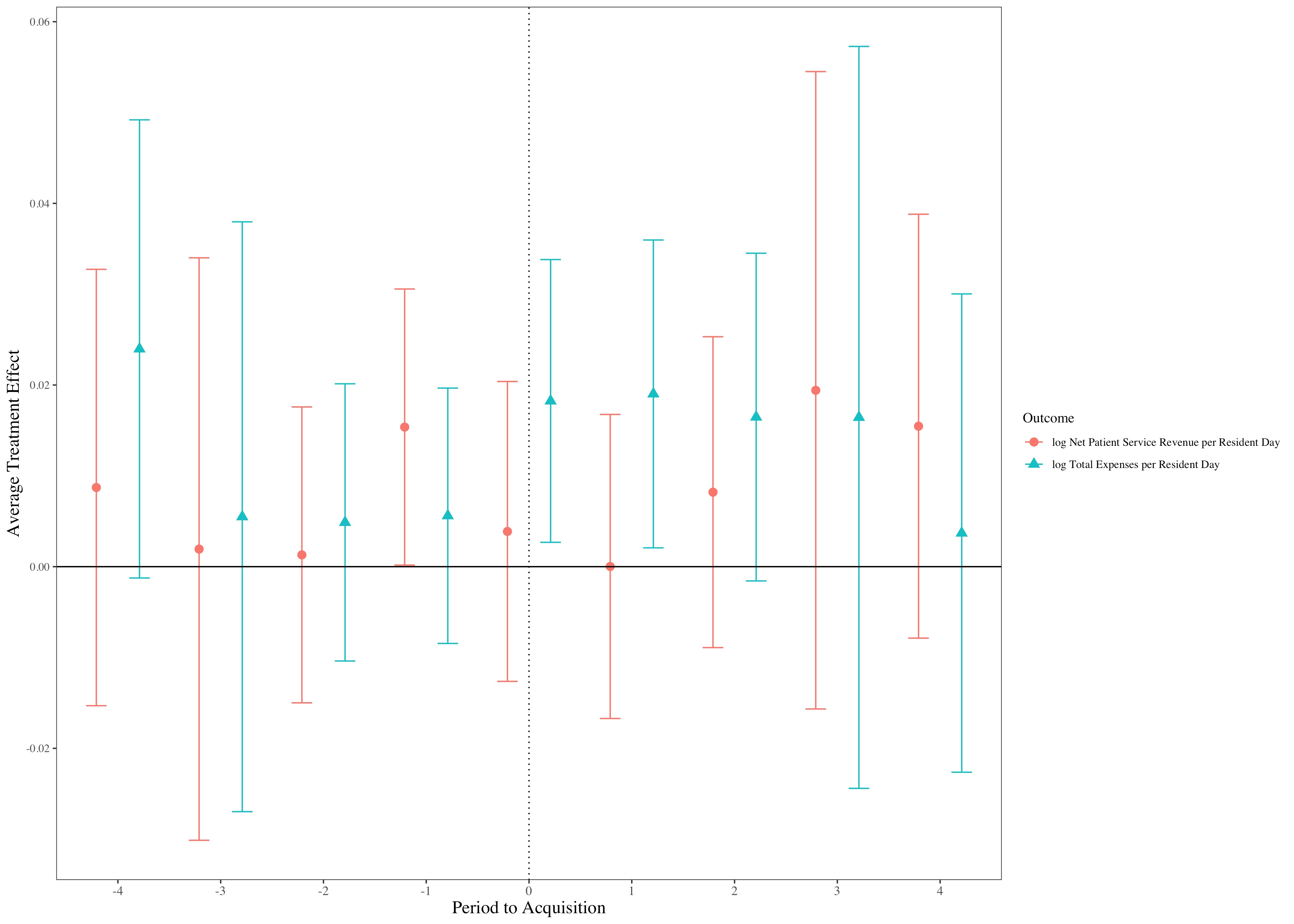


## Appendix 4 Figure 3 – Association of REIT Investment on Nursing Home Total Wages and Total Nursing Wages by Year, 2011-2019


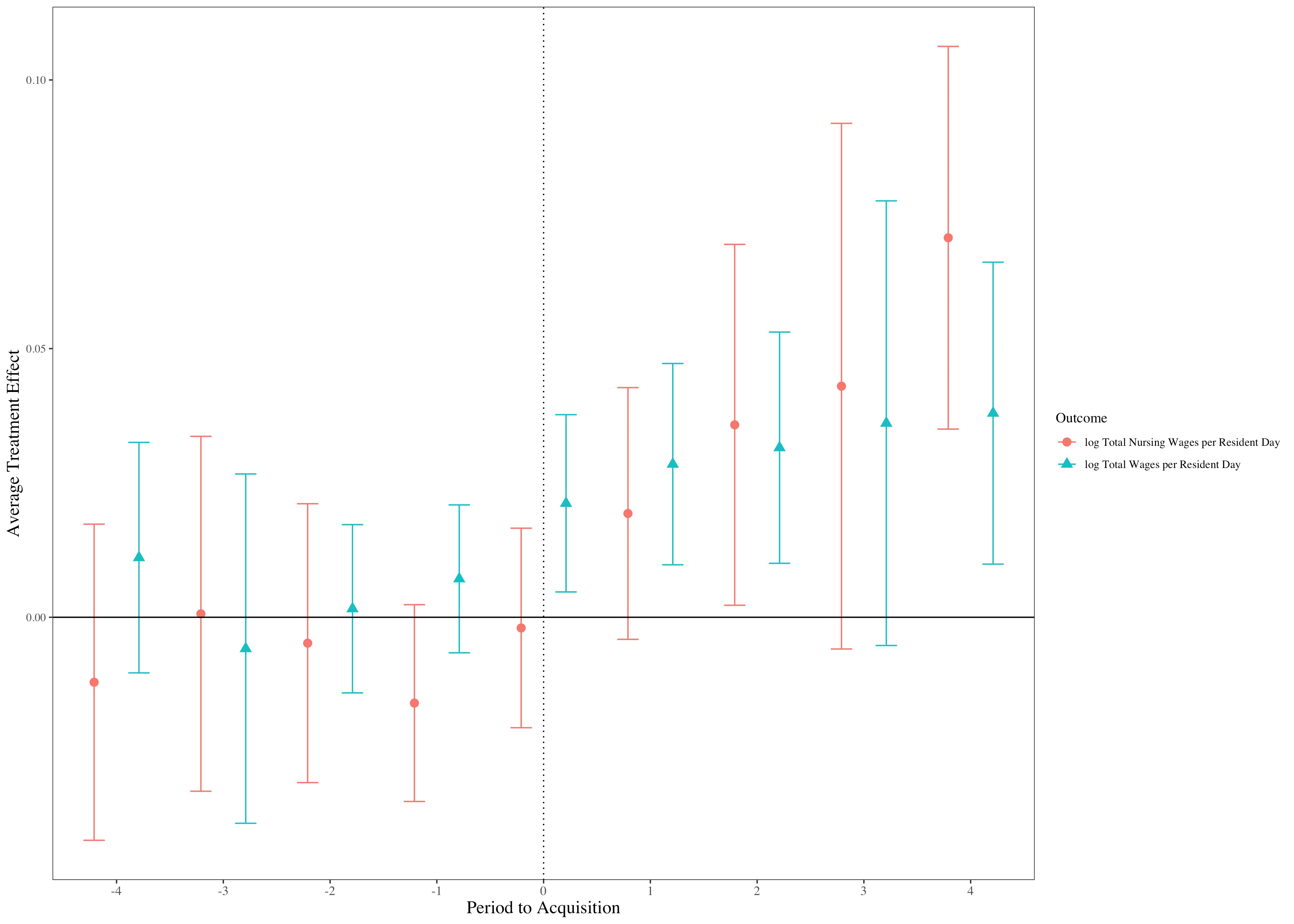


## Appendix 4 Figure 4 – Association of REIT Investment on Nursing Home Current Ratio by Year, 2011-2019


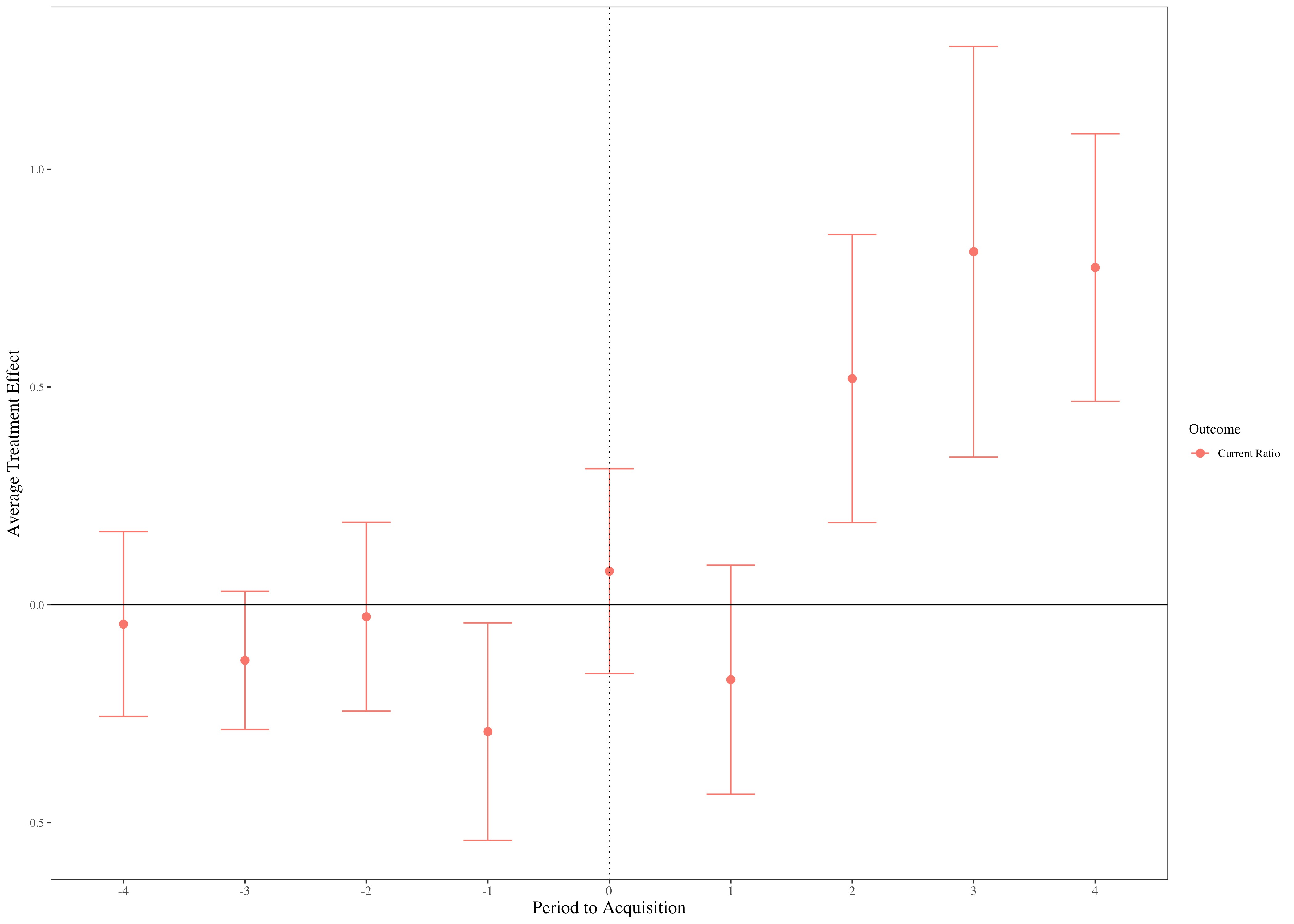


# Appendix 5 – Sensitivity Analyses for Additional Outcomes

Additionally, we evaluated the following outcomes: specific nursing wages

(e.g., registered nursing (RN), licensed practical nursing (LPN), and certified nursing assistants (CNA)) an additional measure of liquidity, days cash on hand (DCOH), and a measure of debt structure, long-term debt to capital (LTDTC).

DCOH was calculated as (((cash + short-term investments) / cash expenses) % 365). While the current ratio is calculated using only balance sheet components, the evaluation of cash expenses in DCOH may make it a more sensitive liquidity measure. Debt structure was measured by LTDTC. LTDTC was calculated as (total long-term liabilities / ( total long-term liabilities + total fund balances)). LTDTC may represent an organization’s longer-term optimal capital structure mix of debt and equity financing. No DID or event study estimates for DCOH or LTDTC were statistically significant (results not presented).

Except for current ratio in REIT-invested facilities (evaluated in the main manuscript), liquidity and capital structure measures were not significantly affected. Although we anticipated some influence on NH capital structure due to the capital influx from REIT or PE investments, and possible subsequent debt reduction, we did not observe any significant correlation with LTDTC. While we did not see significant changes in our debt structure measure, future work would benefit this field of study.

Unadjusted NH nursing wage characteristics are presented in Appendix 5 Table 1. PE NHs had the highest RN, LPN, and CNA wages (all PRD), followed by REIT then other for-profit NHs.

DID results for REIT and PE investments are shown in Appendix 5 Table 2. REIT investment was associated with higher RN wages (5%) and CNA wages (4%) (both logged, PRD). There were no other statistically significant associations in the DIDs.

In event study plots, REIT investment was associated with higher RN wages (year 4, Appendix 5 Figure 1), LPN wages (year 2), and CNA wages (years 2 – 4).

There were no statistically significant associations for PE investment in NHs in the DIDs or event study plots for these measures.

## Appendix 5 Table 1 – Additional Characteristics of Nursing Homes by For-Profit (Control), REIT, or PE Association in the Pooled Sample, 2011-2019

| **Appendix 5 Table 1** – **Additional Characteristics of Nursing Homes by For-Profit (Control), REIT, or PE Association in the Pooled Sample (2011 – 2019)** | | | |
| --- | --- | --- | --- |
|  | **2011-2019 Pooled Sample** | | |
| **Characteristics** | **For-Profit (n=5,138)** | **REIT (n=301)** | **PE (124)** |
| **RN Wage Costs per Resident Day, mean** | **$15.78** | **$19.04** | **$24.27** |
| **LPN Wage Costs per Resident Day, mean** | **$21.58** | **$23.64** | **$23.58** |
| **CNA Wage Costs per Resident Day, mean** | **$32.09** | **$33.53** | **$34.42** |
| **Total Contract Nurse Wage Costs per Resident Day, mean** | **$1.89** | **$1.45** | **$2.87** |

Source: Authors’ analysis of Cost Report and LTCFocus data from 2011-2019.

Notes: ***p<0.01, **p<0.05, *p<0.1

REIT (Real estate investment trust)

For-profit includes nursing homes that never received REIT or PE investment during the sample period.

We excluded homes where we determined REITs or PE divested before 2018, invested before 2012, or invested after 2018.

## Appendix 5 Table 2 – Sensitivity Analyses for Difference-in-Differences in Additional Outcomes After REIT or PE Investment Compared to For-Profit Nursing Homes without PE or REIT Ownership, 2011-2019

| **Appendix 5 Table 2 – Difference-in-Differences in Additional Outcomes After REIT or PE Investment Compared to For-Profit Nursing Homes without PE or REIT Ownership** | | | | |
| --- | --- | --- | --- | --- |
|  |  | **REIT (n = 2507)** |  | **PE (n = 977)** |
| **Outcome** | **REIT Pooled Sample, 2011-2019** | **ATT** | **PE Pooled Sample, 2011-2019** | **ATT** |
| log RN Wage Costs per Resident Day | 15.99 | 0.05* | 17.74 | 0.06 |
| log LPN Wage Costs per Resident Day | 21.72 | 0.01 | 22.66 | -0.01 |
| log CNA Wage Costs per Resident Day | 32.18 | 0.04*** | 33.71 | -0.01 |
| log Total Contract Nurse Wage Costs per Resident Day | 1.86 | 0.22 | 2.27 | 0.21 |

Source: Authors’ analysis of Cost Report and LTCFocus data from 2011-2019.

Notes: ***p<0.01, **p<0.05, *p<0.1

DD (Difference-in-Differences)

Estimates generated from Callaway and Sant’Anna (2021) difference-in-differences estimator. Implements a difference-in-difference with multiple periods estimator to decompose a two-way fixed effects (TWFE) model with staggered treatment to individual 2x2 difference-in-differences estimations.

ATT: Average treatment effect on treated.

Per Resident Day (PRD) was calculated by dividing the outcome dollars by 365 days.

## Appendix 5 Figure 1 – Association of REIT Investment on Nursing Home RN, LPN, and CNA Wages by Year, 2011-2019


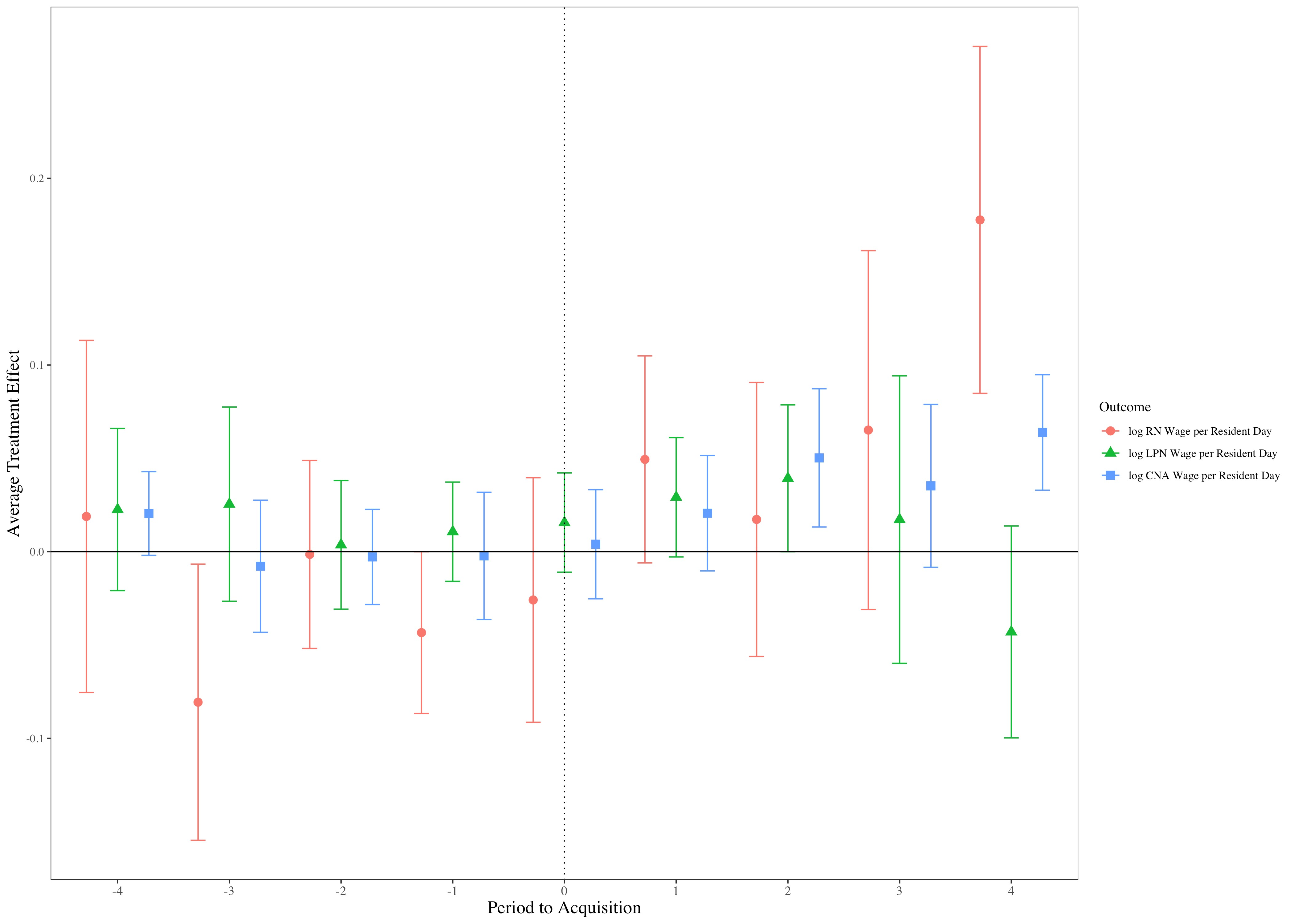


# Appendix 6 – Sensitivity Analyses for Selection Based on Resident and Facility Characteristics

| **Appendix 6 – Selection Based on Patient and Facility Characteristics for Changes in Financial Outcomes After Investment Compared to For-Profit Nursing Homes without PE or REIT Ownership** | | | |  |
| --- | --- | --- | --- | --- |
|  |  | **REIT (n = 2507)** |  | **PE (n = 977)** |
| **Outcome** | **REIT Pooled Sample, 2011-2019** | **ATT** | **PE Pooled Sample, 2011-2019** | **ATT** |
| Average Age | 78.62 | -0.18 | 78.92 | -0.03 |
| Percentage Female | 65.76 | -0.34 | 66.04 | -1.02* |
| Percentage Medicaid | 62.58 | 1.61** | 64.05 | 2.78** |
| Percentage Medicare | 14.36 | -0.32 | 14.61 | -1.26 |
| Percentage White | 78.62 | -0.50 | 76.60 | 1.46* |
| Total Beds | 108.19 | 0.87 | 110.55 | 0.67 |
| Occupancy Percent | 79.72 | 0.92 | 83.34 | 0.46 |
| Acuity Index | 12.08 | 0.06 | 12.32 | 0.15 |

Source: Authors’ analysis of Cost Report and LTCFocus data from 2011-2019.

Notes: ***p<0.01, **p<0.05, *p<0.1

DD (Difference-in-Differences)

Estimates generated from Callaway and Sant’Anna (2021) difference-in-differences estimator. Implements a difference-in-difference with multiple periods estimator to decompose a two-way fixed effects (TWFE) model with staggered treatment to individual 2x2 difference-in-differences estimations.

ATT: Average treatment effect on treated.

Per Resident Day (PRD) was calculated by dividing the outcome dollars by 365 days.

# Appendix 7 – Sensitivity Analyses Accounting for Year and State Fixed Effects

| **Appendix 7. Sensitivity Analyses Accounting for Year and State Fixed Effects, Changes in Financial Outcomes After PE Firm Investment Compared to For-Profit Nursing Homes without PE or REIT Ownership** | | | | |
| --- | --- | --- | --- | --- |
|  |  | **REIT (n = 2507)** |  | **PE (n = 977)** |
| **Outcome** | **REIT Pooled Sample, 2011-2019** | **ATT** | **PE Pooled Sample, 2011-2019** | **ATT** |
| Total Margin | 0.59 | -0.65 | 0.54 | 0.41 |
| Operating Margin | -0.44 | -0.80 | -0.68 | 0.27 |
| log Net Patient Service Revenue per Resident Day | 280.42 | 0.00 | 298.31 | -0.07*** |
| log Total Salaries per Resident Day | 122.96 | 0.03*** | 129.65 | -0.08*** |
| log Total Expenses per Resident Day | 290.10 | 0.01 | 310.05 | -0.06*** |
| log Total Nurse Wage Costs per Resident Day | 69.31 | 0.02** | 73.61 | 0.00 |
| log RN Wage Costs per Resident Day | 15.99 | 0.06** | 17.74 | 0.07 |
| log LPN Wage Costs per Resident Day | 21.72 | -0.01 | 22.66 | 0.01 |
| log CNA Wage Costs per Resident Day | 32.18 | 0.02** | 33.71 | -0.03 |
| log Total Contract Nurse Wage Costs per Resident Day | 1.86 | 0.43 | 2.27 | 0.24 |
| Current Ratio | 2.01 | 0.75*** | 1.97 | 0.14 |

Source: Authors’ analysis of Cost Report and LTCFocus data from 2011-2019.

Notes: ***p<0.01, **p<0.05, *p<0.1

DD (Difference-in-Differences)

Estimates generated from Callaway and Sant’Anna (2021) difference-in-differences estimator. Implements a difference-in-difference with multiple periods estimator to decompose a two-way fixed effects (TWFE) model with staggered treatment to individual 2x2 difference-in-differences estimations.

ATT: Average treatment effect on treated.

Per Resident Day (PRD) was calculated by dividing the outcome dollars by 365 days.

# Appendix References

1. Rivest LP. Statistical Properties of Winsorized Means for Skewed Distributions. Biometrika. 1994;81(2):373-83.

2. Williams Jr. D, Holmes GM, Song PH, Reiter KL, Pink GH. For Rural Hospitals That Merged, Inpatient Charges Decreased and Outpatient Charges Increased: A Pre-/Post-Comparison of Rural Hospitals That Merged and Rural Hospitals That Did Not Merge Between 2005 and 2015. The Journal of Rural Health. 2020;n/a(n/a).

3. Bowblis JR. The Need for an Economically Feasible Nursing Home Staffing Regulation: Evaluating an Acuity-Based Nursing Staff Benchmark. Innovation in Aging. 2022;6(4).

4. CMS.gov. Medicare and Medicaid Programs: Minimum Staffing Standards for Long-Term Care Facilities and Medicaid Institutional Payment Transparency Reporting (CMS 3442-P). Sep 01, 2023. <https://www.cms.gov/newsroom/fact-sheets/medicare-and-medicaid-programs-minimum-staffing-standards-long-term-care-facilities-and-medicaid#:~:text=The%20proposed%20rule%20consists%20of,and%203)%20enhanced%20facility%20assessment>.

5. Author's analysis of Medicare Cost Report data for relevant years.
